# β-Arrestin 2 Promotes Hepatocyte Apoptosis by Inhibiting Akt Pathway in Alcoholic Liver Disease

**DOI:** 10.3389/fphar.2018.01031

**Published:** 2018-09-19

**Authors:** Ying-Yin Sun, Yu-Xin Zhao, Xiao-Feng Li, Cheng Huang, Xiao-Ming Meng, Jun Li

**Affiliations:** Anhui Key Laboratory of Bioactivity of Natural Products, School of Pharmacy, Anhui Medical University, Hefei, China

**Keywords:** alcoholic liver disease (ALD), β-arrestin 2 (Arrb2), hepatocyte, apoptosis, Akt

## Abstract

Alcoholic liver disease (ALD) is a complex process that includes a wide range of hepatic lesions, from steatosis to cirrhosis, and even hepatocellular carcinoma (HCC). Accumulating evidence shows that the cytotoxic effects of ethanol metabolism lead to cell apoptosis and necrosis in ALD. Recently, several studies revealed that multifunctional protein β-arrestin 2 (Arrb2) modulated cell apoptosis in liver fibrosis and HCC, but its role in ALD has not been fully understood. The aim of this study is to explore the function and underlying mechanism of Arrb2 in hepatocyte survival and apoptosis in ALD. In our study, the primary hepatocytes were isolated from the livers of C57BL/6 mice fed EtOH-containing diet, it showed an increased level of Arrb2. EtOH also significantly up-regulated Arrb2 production in AML-12 cells *in vitro*. Furthermore, TUNEL (terminal deoxynucleotidyl transferase-mediated dUTP nick end labeling) and FCM results demonstrated that knockdown of Arrb2 could inhibit hepatocyte apoptosis induced by EtOH *in vivo* and *vitro* while over-expression of Arrb2 induced apoptosis in ALD. In addition, western blot results revealed that Arrb2 remarkably suppressed the Akt signaling. Taken together, our data suggested that Arrb2 may serve as a potential therapeutic target for ALD by promoting hepatocyte apoptosis via Akt suppression.

## Introduction

Alcoholic liver disease (ALD) is a complex disease that becomes one of the leading cause of severe liver-related morbidity and significant mortality worldwide.

Every year, about 3.3 million deaths occur in worldwide because of the prolonged alcohol abuse according to the World Health Organization (Arsene et al., 2016). ALD includes a wide spectrum of hepatic lesions, from steatosis, alcoholic steatohepatitis, progressive fibrosis, cirrhosis to even hepatocellular carcinoma (HCC) due to a consequence of susceptibility factors and degree of alcohol consumption (Ju and Mandrekar, 2015).

So far, the pathogenic mechanisms of ALD include the direct cytotoxic effect of alcohol and its metabolites like acetaldehyde, they may induce oxidative stress in hepatocytes and finally lead to cell inflammation, injury, and death (Louvet and Mathurin, 2015). Accumulating evidence demonstrated that apoptosis of massive hepatocytes is a prominent feature of the initiation and progression stages of ALD (Verma et al., 2016). In this regard, inhibition of apoptotic hepatocytes is critical in relieving the degree of ALD, and it is essential to find a potential therapeutic target for treating disease (Wu et al., 2016).

β-Arrestin, also termed 48-kDa protein, was found in 1986 as a cytosolic protein initially (Wilden et al., 1986). It not only serves as a co-factor in restraining the process of the light recept or rhodopsin to photoactivation by rhodopsin kinase but also suppresses the activation of cGMP phosphor-diesterase in retinal rod disk membranes (Kingsmore et al., 1995). There are four members in the arrestin family. Arrestin 1 and 4, which called the visual arrestins, can be found in the visual system exclusively, whereas the arrestins 2 and 3 (also called β-arrestin 1 and 2) are confirmed to exist in mammalian tissue ubiquitously (Barki-Harrington and Rockman, 2008).

It is well documented that multifunctional adaptor β-arrestin 2 (Arrb2) modulates cell apoptosis and it may have either a pro- or anti-apoptotic effect in different diseases. For instance, Arrb2 promotes hepatocyte apoptosis in bile duct ligation (BDL) while blocking hepatic stellate cells (HSCs) apoptosis in liver fibrosis (Sun et al., 2013; Yin et al., 2016; Zhang et al., 2017). However, the role of Arrb2 in modulating apoptosis during ALD remains unclear.

In this study, our data showed that Arrb2, but not Arrb1, significantly induced hepatocyte apoptosis in ALD by inhibiting Akt signaling pathway. Arrb2 depletion could inhibit hepatocyte apoptosis induced by EtOH both *in vivo* and *vitro*. Taken together, our data suggested Arrb2 plays a critical role in ALD via Akt signal, it may be a potential therapeutic target for ALD.

## Materials and Methods

### Animal, Mouse Model of ALD

Eight-week-old male C57BL/6J mice were provided by the Experimental Animal Center of Anhui Medical University. All the animal experiments were approved by the Ethics Committees of Anhui Medical University and all procedures were performed under the permission of the Guideline of Animal Care and Use Committee of Anhui Medical University. For all experiments, mice were divided randomly into four groups, CD-fed + pGLV2-NC, EtOH-fed + pGLV2-NC, CD-fed + pGLV2-Arrb2, EtOH-fed + pGLV2-Arrb2, and all mice were fed at comfortable environment for at least 1 week. Modeling process of Gao-Binge protocol has a total of 16 days including adaptation period for 5 days and modeling for 10 days with 5% v/v ethanol liquid diets, EtOH-fed mice were gavaged with one time binge ethanol administration (5 g/kg, body weight, 20% ethanol) at the last day (Bertola et al., 2013). Mice were injected with lentivirus in caudal vein at the beginning and the middle of the modeling period. All mice were anesthetized after 9 h since the last time of gavage alcohol. The mice blood and liver tissue were separated for preparing further experiments.

### Isolation of Liver Hepatocytes

Isolated liver hepatocytes were perfused from liver tissue of mice by using collagenase (type I; Sigma-Aldrich, St. Louis, MO, United States) perfusion. We referred to the previous papers to find the perfusion methods (Smedsrod and Pertoft, 1985; Hansen et al., 2002; Cassim et al., 2017). First, cannulating the cannula and then cutting out inferior vena cava. Next, perfusing the liver with 1% EGTA solution [1 × EGTA, NaCl (80 g), KCl (4 g), KH_2_PO_4_ (0.6 g), NA_2_HPO_4_ (0.48 g) and EGTA (1.9 g) in H_2_O (1000 ml)], and then via recirculation with collagenase until the hepatic parenchyma appeared liquefied. Afterward, removing and placing liver in a sterile dish and add digestion buffer [0.075% collagenase 3 ml and 24 ml GBSS (Gey’s Balanced Salt Solution)]. Cutting liver into very small pieces and shaking for 30 min at 200 rpm in 37°C incubator. Finally, adding GBSS and then centrifuging 400 rpm for 5 min to collect hepatocytes for further RNA and protein analysis.

### Cell Culture and Treatment

AML-12 cells were obtained from American Type Culture Collection (ATCC) (Shanghai, China), cells were cultured in F-12 medium (Gibco, United States) supplemented with 8% fetal bovine serum (Clark, United States) and incubated at 37°C at an atmosphere of 5% CO_2_. AML-12 cells were cultured in F-12 medium with EtOH for 24 h while the non-treated AML-12 cells were used as control (Aller de la Fuente et al., 2018).

### Histopathological Examination and Immunofluorescent Staining

For histologic analysis, liver tissues were fixed with 10% neutral formalin solution and dehydrated with different concentration of ethanol. After then treated with xylene and embedded in paraffin. The paraffin liver tissue were cut into 5 μm thick sections and stained with hematoxylin and eosin (H & E). For immunofluorescent staining, liver tissues were fixed with 4% paraformaldehyde and then blocked with 10% BSA for 10 min. To investigate the expression of Arrb2 in mice liver, the tissue were incubated with mouse anti-Arrb2 antibody (1:100) overnight at 4°C and anti-mouse FITC (1:200) at temperature for 2 h. At last, the section was mounted with DAPI and images were taken using fluorescence microscopy.

### The ALT/AST Activity Assay and the Serum Levels of TG/TCH Analysis

The serum levels of ALT, AST, TG, and TCH in mice with alcohol-induced ALD were assayed by using alanine aminotransferases (ALT) assay kit, aspartate aminotransferases (AST) assay kit, triglyceride (TG) and total cholesterol (TCH) assay kits. All kits were from Nanjing Jiancheng Bioengineering Institute. The absorbance was measured with a micro-plate reader model 680 (Bio-Rad Laboratories, Hercules, CA, United States).

### Flow Cytometry Analysis

The level of Apoptosis was quantified by FITC-Annexin V apoptosis detection kit (BestBio, China). Firstly, AML-12 cells were washed by cold PBS three times. Then cells sedimentation were resuspended in binding buffer at a density of 1 × 10^6^/ml. Next, adding 10 μl PI and 5 μl Annexin V-FITC to stain apoptosis cells. Staining cells were calculated with BD LSR flow cytometer (BD Biosciences, San Jose, CA, United States) and the data was analyzed by a software named FlowJo (Gondhalekar et al., 2018; Libregts et al., 2018).

### TUNEL Staining

To visualize apoptotic bodies, cells slides were firstly fixed in 10% buffered formalin at room temperature for 25 min and then supplemented with 0.2% Triton X-100 solution in PBS after washing twice with PBS at room temperature for 5 min. Subsequently, cells slides were covered with 100 μl equilibration buffer and then equilibrated for 10 min. Add rTdT reaction mix to the slides and the slide was covered with coverslip. Next, remove the coverslip and terminate the reaction using saline-sodium citrate. After that, cell slides were immersed in 0.3% H_2_O_2_ for 3–5 min. After being washed three times with PBS, slides were immersed in 50 μl of TUNEL (terminal deoxynucleotidyl transferase-mediated dUTP nick end labeling) detection solution at 15–25°C for 60 min. At last, slides were incubated with DAPI (Bi Yuntian Biological Technology, China) for 10 min. TUNEL positive cells were visualized with a fluorescence microscope.

### Small Interfering RNA Analysis

According to the manufacturer’s protocol, transfection of AML-12 cells was carried out with 100 nM of small interfering RNA (siRNA) by using Lipofectamine^TM^ 2000 (Invitrogen, Carlsbad, CA, United States). The Arrb2-siRNA and a negative scrambled siRNA were both synthesized by GenePharma (Shanghai, China). The siRNA sequences were as follows: siRNA-Arrb2 (mouse), 5′-GGACCAGGGUCUUCAAGAATT-3′ (sense) and 5′-UUCUUGAAGACCCUGGUCCTT-3′ (antisense). The AML-12 cells were cultured in F-12 for 12 h, and then subjected to reverse transfection by using Opti-MEM (Gibco, United States). Next, the culture medium was changed after 6 h transfection. Quantitative real-time PCR and western blot analysis were used after siRNA transfection. It was worth noting that all experiments were repeated three times.

### Plasmid Construction

The mouse pEX3-Arrb2 was purchased from GenePharma (Shanghai, China). Using pEX3-Arrb2 transfection made ectopic high expression of Arrb2 and empty vector pEX3 was used as control. Firstly, the constructed plasmid was transfected into AML-12 cells and then using quantitative real-time PCR and western blot for further analysis. All experiments were repeated three times in the same way.

### Total RNA Extraction and Quantitative Real-Time PCR

Total RNA was extracted from liver hepatocyte and AML-12 cells by using TRIzol (Invitrogen, United States), and then reverse transcribed to the first-strand cDNA by using TaKaRa kit (QIAGEN, Japan). The mRNA expression of Arrb2 was detected by quantitative real-time PCR analyses kits (Applied Biosystems, United States). The mRNA level of β-actin was used as an internal control. Quantitative real-time PCR was performed at 95°C for 10 min followed by 40 cycles at 95°C for 15 s and at 60°C for 1 min. The primers sequences were listed as follows: Arrb2 (forward: GGCAAGCGCGACTTTGTAG and reverse: GTGAGGGTCACGAACACTTTC).

### Western Blot Analysis

Isolated mouse hepatocyte and AML-12 cells were lysed with RIPA lysis buffer containing PMSF (100:1). The concentration of extract protein was determined using BCA protein assay kit (Beyotime, Jiangsu, China). Equal amounts of extracted protein were separated by SDS-PAGE and blotted onto PVDF membranes. Firstly, block non-specific binding with TBST containing 5% skim milk for 1 h at room temperature. Then nitrocellulose blots were incubated with the primary antibody against Arrb2 for 12 h at 4°C. The next day, the membranes were incubated with HRP-conjugated secondary antibodies at 37°C for 1 h after washed three times with TBS/Tween 20 (0.075%). Signals of bands were visualized with ECL-chemiluminescent kit. The characteristics of antibodies were listed in **Supplementary Table 1**.

### Statistical Analysis

All data were presented as means ± SD analyzed using Statistical Package for Social Sciences (SPSS Inc., Chicago, IL, United States, version 13.0). Two samples were analyzed by using *t*-test, multiple samples were analyzed by using Kruskal–Wallis one-way analysis of variance (ANOVA). In all cases, *P* < 0.05 was considered statistically significant.

## Results

### Pathological Characteristics and Characterization of a Mouse Model of ALD After Binge Ethanol Feeding

**FIGURE 1:**
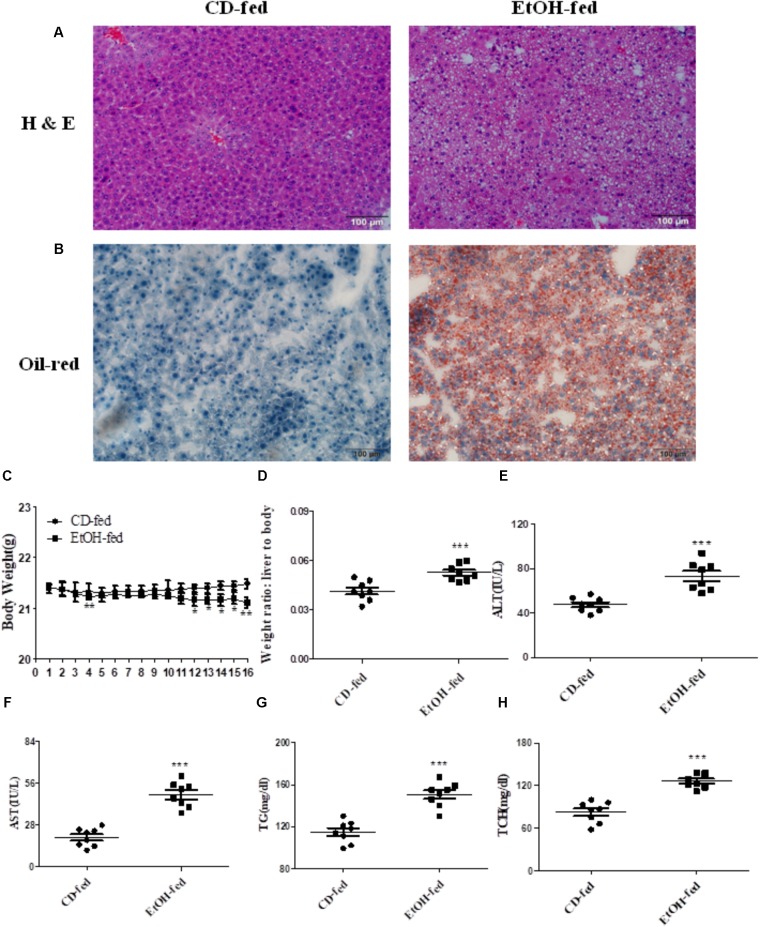
Pathological characteristics in ALD mouse model after binge ethanol feeding. **(A)** Representative views of hematoxylin and eosin (H & E) staining of liver tissues (original magnification, ×20). **(B)** Representative views of Oil Red O staining of liver tissues (original magnification, ×20). **(C)** Body weights after ethanol feeding. **(D)** The liver to body weight radio after ethanol feeding. **(E)** The serum levels of ALT. **(F)**The serum levels of AST. **(G)** Hepatic triglyceride (TG) levels. **(H)** Hepatic total cholesterol (TCH) levels. The values represent means ± SD. (*n* = 6 in CD-fed group, *n* = 6 in EtOH-fed group) ^∗^*P* < 0.05, ^∗∗^*P* < 0.01, ^∗∗∗^*P* < 0.001 vs. CD-fed group.

As described in Section “Materials and Methods,” all the male C57BL/6J mice fed with alcohol for 16 days displayed significant immune cell inflammation, injury and plenty of lipid accumulation in the liver, while the CD-fed mice showed normal cell state. The degree of liver injury and histological features were assessed by using hematoxylin eosin (H & E) staining and Oil Red O staining. As shown in **Figure 1A**, liver tissues in EtOH-fed mice displayed liver cell cord derangement, fat lipid vacuoles, cell spaces dilatation, and inflammatory cell infiltration compared to the CD-fed mice that showed normal radiating hepatic cord. Moreover, the liver tissue of EtOH-fed mice exhibited abundant lipid droplet by using Oil Red O staining (shown in **Figure 1B**). In the initial stage, body weights of both EtOH-fed and CD-fed mice were decreased slightly. After a short-time adaptive phase, body weight in both group was gradually increased, but the body weight of EtOH-fed mice was significantly decreased at the end of the stage and still lower than the beginning (**Figure 1C**). Interestingly, the liver to body weight ratio of EtOH-fed group was notably higher than CD-fed group (**Figure 1D**). The serum levels of ALT and AST in EtOH-fed mice was remarkably increased compared to the CD-fed mice (**Figures 1E,F**). The metabolic changes in the above result were further confirmed by measuring the degree of TG and TCH (**Figures 1G,H**).

### Expression Level of Arrb2 in Mouse Liver Is Significantly Increased After Binge Ethanol Feeding

**FIGURE 2:**
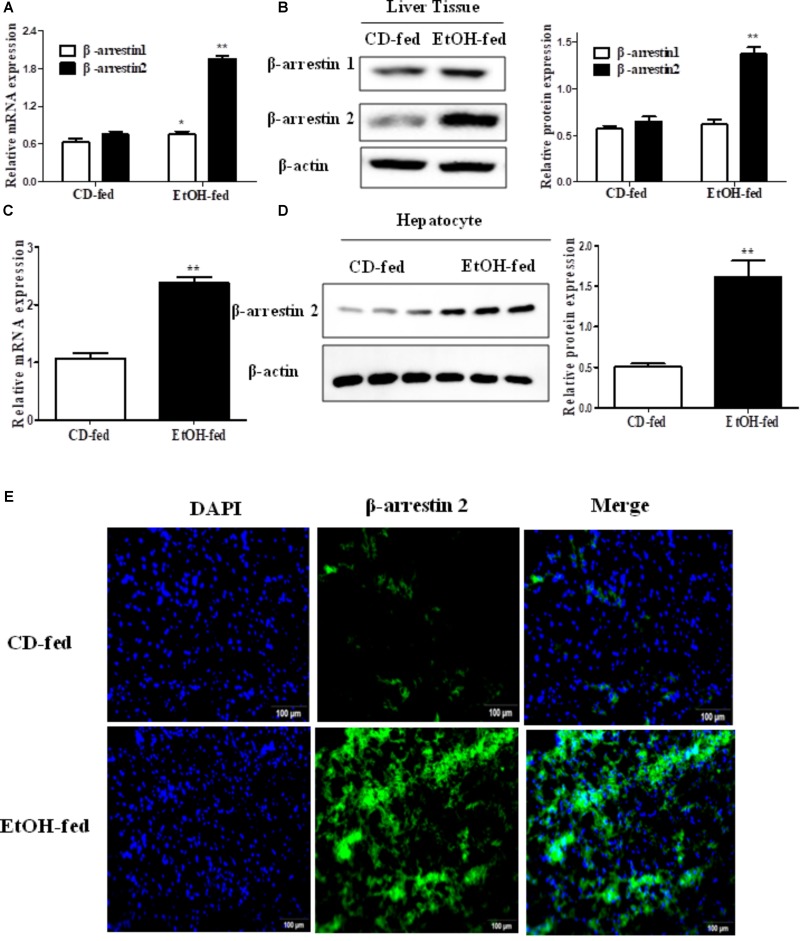
Effect of alcohol on Arrb2 expression in liver tissues and hepatocytes in ALD mouse model. **(A)** Total Arrb1 and Arrb2 mRNA levels in liver tissues were analyzed by real-time PCR. **(B)** The protein levels of Arrb1 and Arrb2 in liver tissues were analyzed by western blot in ALD mouse model. **(C)** Total Arrb2 mRNA levels in hepatocytes isolated from liver tissues were analyzed by real-time PCR. **(D)** The protein levels of Arrb2 in hepatocytes isolated from liver tissues were analyzed by western blot. **(E)** The expression of Arrb2 in liver tissues was analyzed by immunofluorescence (IF) assay (original magnification, ×20). The values represent means ± SD. (*n* = 6 in CD-fed group, *n* = 6 in EtOH-fed group) ^∗^*P* < 0.05, ^∗∗^*P* < 0.01, ^∗∗∗^*P* < 0.001 vs. CD-fed group.

To investigate the expression profile of Arrb1 and Arrb2 between the EtOH-fed group and CD-fed group, real-time PCR and western blot were used to detect the mRNA level and protein level of liver tissues, respectively. As shown in **Figures 2A,B**, both mRNA levels of Arrb1 and Arrb2 were increased, and the upregulation of Arrb2 is more significant than Arrb1. So we presumed that Arrb2 may play more important role in ALD. Next, we detected the expression of Arrb2 in the hepatocytes isolated from the mice liver. mRNA and protein level of Arrb2 were increased by more than onefold compared with CD-fed group (**Figures 2C,D**). Immunofluorescent (IF) analysis further showed the changes of Arrb2 and the results in **Figure 2E** showed a prominent increase in EtOH-fed group mice.

### Arrb2 Induces Hepatocytes Apoptosis *in vivo*

**FIGURE 3:**
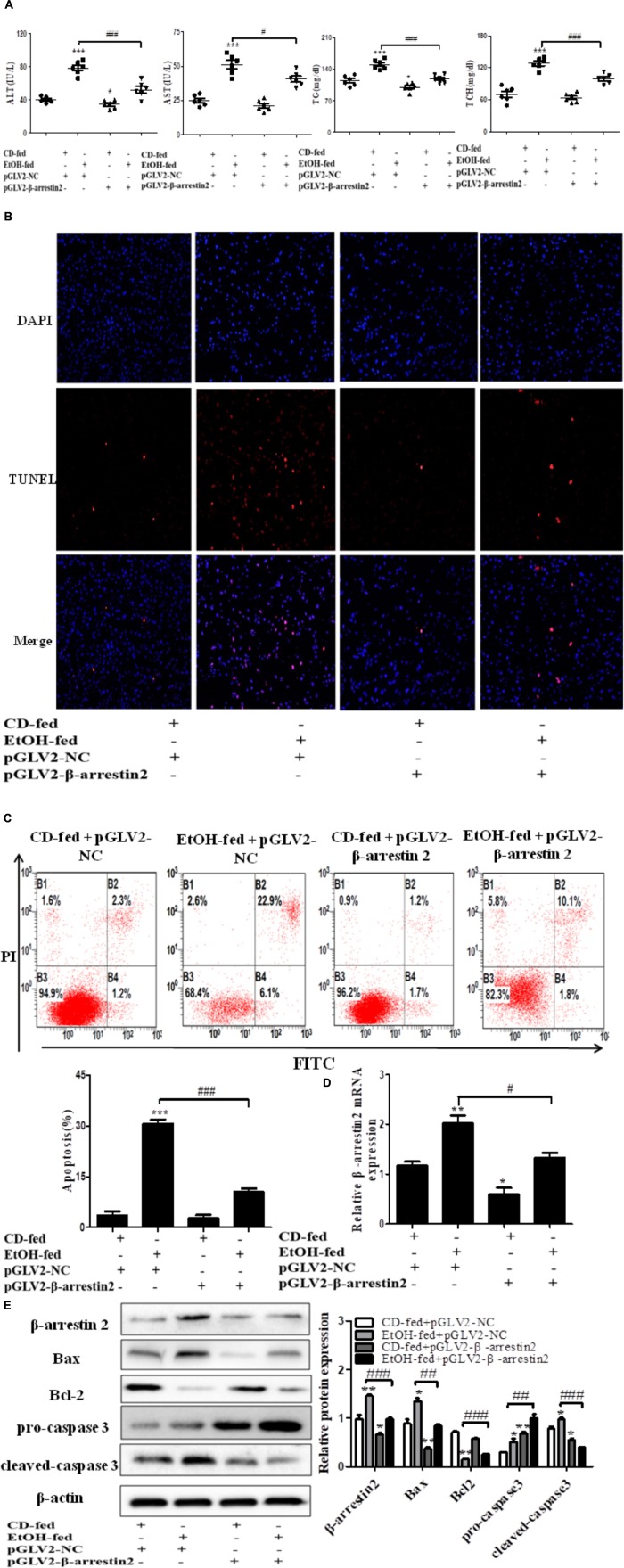
Effect of Arrb2 silencing on ALD mouse model in hepatocytes. **(A)** The serum levels of ALT, AST, TG, TCH were detected after injecting lentivirus to knockdown Arrb2 expression. **(B)** Representative images of TUNEL staining in different groups by using fluorescence microscope (original magnification, ×20). **(C)** Apoptosis of hepatocytes isolated from ALD model mice liver tissues were analyzed by flow cytometry with Annexin V-FITC and PI staining. **(D)** The mRNA levels of Arrb2 were detected by real-time PCR. **(E)** The apoptosis relative protein expression were observed by western blot. The results are shown as relative expression against control expression without treatment. The values represent means ± SD. (*n* = 3 in CD-fed group, *n* = 3 in EtOH-fed group). Data shown are the mean ± SD from three independent experiments. ^∗^*P* < 0.05, ^∗∗^*P* < 0.01, ^∗∗∗^*P* < 0.001 vs. CD-fed + pGLV2-NC. ^#^*P* < 0.05, ^##^*P* < 0.01, ^###^*P* < 0.001 vs. EtOH-fed + pGLV2-NC.

The observation above indicated that up-regulation of Arrb2 may play key roles in the progression of ALD. Hence, to explore whether Arrb2 affects the development of ALD by regulating hepatocytes apoptosis, the lentivirus were used to knockdown the expression of Arrb2 in ALD mouse model. The lentivirus vector have fluorescence and glow green by using microscope. Firstly, the changes of ALT, AST, TG, and TCH in serum were detected to evaluate the effect of Arrb2 on alcoholic liver injury. The results in **Figure 3A** showed that the degree of liver injury was decreased in pGLV2-Arrb2 mice group. Secondly, the TUNEL staining and apoptosis assay by flow cytometric analysis were carried out. As shown in **Figure 3C**, the sum of B2 and B4 quadrant fraction displayed apoptotic cells. Therefore, the results in **Figures 3B,C** demonstrated that down-regulated expression of Arrb2 could suppress hepatocyte apoptosis. To further investigate the effect of Arrb2 in regulating hepatocyte apoptosis, real-time PCR, and western blot were used to detect the mRNA levels of Arrb2 after lentivirus injection and apoptosis related proteins levels in pGLV2-Arrb2 mice group. The results in **Figures 3D,E** further indicated that Arrb2 could induce hepatocytes apoptosis *in vivo.*

### Arrb2 Induces Hepatocyte Apoptosis *in vitro*

**FIGURE 4:**
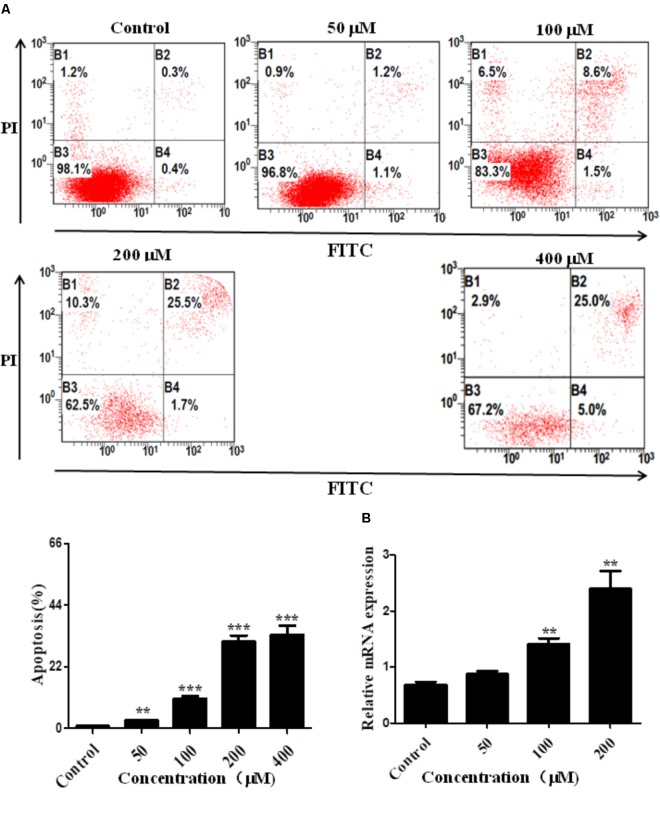
Effect of alcohol on Arrb2 expression *in vitro*. **(A)** Effect of different concentrations of ethanol on AML-12 cells apoptosis were detected by flow cytometry with Annexin V-FITC and PI staining. **(B)** The mRNA levels of Arrb2 at different concentrations were detected by real-time PCR. The results are shown as relative expression against control expression without treatment. ^∗^*P* < 0.05, ^∗∗^*P* < 0.01, ^∗∗∗^*P* < 0.001 vs. Control.

**FIGURE 5:**
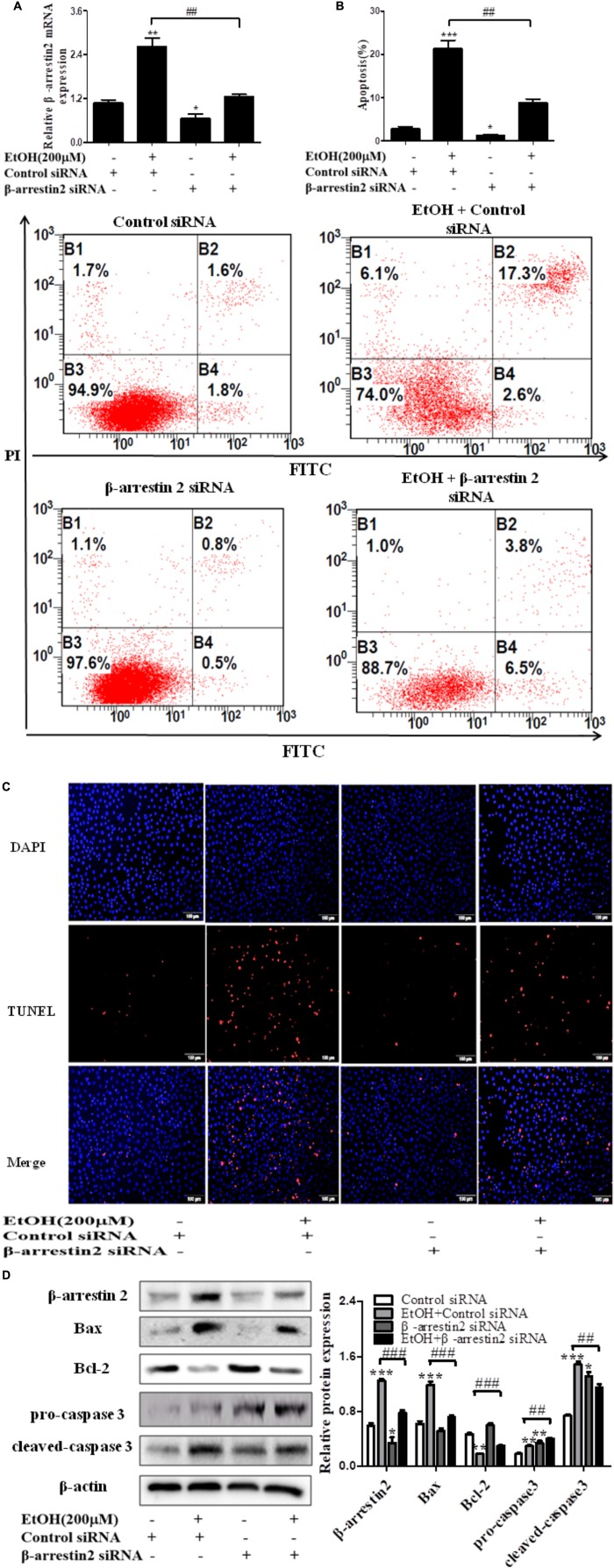
Effect of Arrb2 siRNA on EtOH-stimulated AML-12 cells. **(A)** The Arrb2 levels were detected by real-time PCR after transfection. The results are shown as relative expression against control expression without treatment. **(B)** Apoptosis of AML-12 cells were analyzed by flow cytometry with Annexin V-FITC and PI staining. **(C)** Representative images of TUNEL staining in different groups (original magnification, ×20). **(D)** The apoptosis relative protein expression were detected by western blot. A representative image of the three independent experiments were demonstrated. ^∗^*P* < 0.05, ^∗∗^*P* < 0.01, ^∗∗∗^*P* < 0.001 vs. Control siRNA. ^#^*P* < 0.05, ^##^*P* < 0.01, ^###^*P* < 0.001 vs. EtOH + Control siRNA.

**FIGURE 6:**
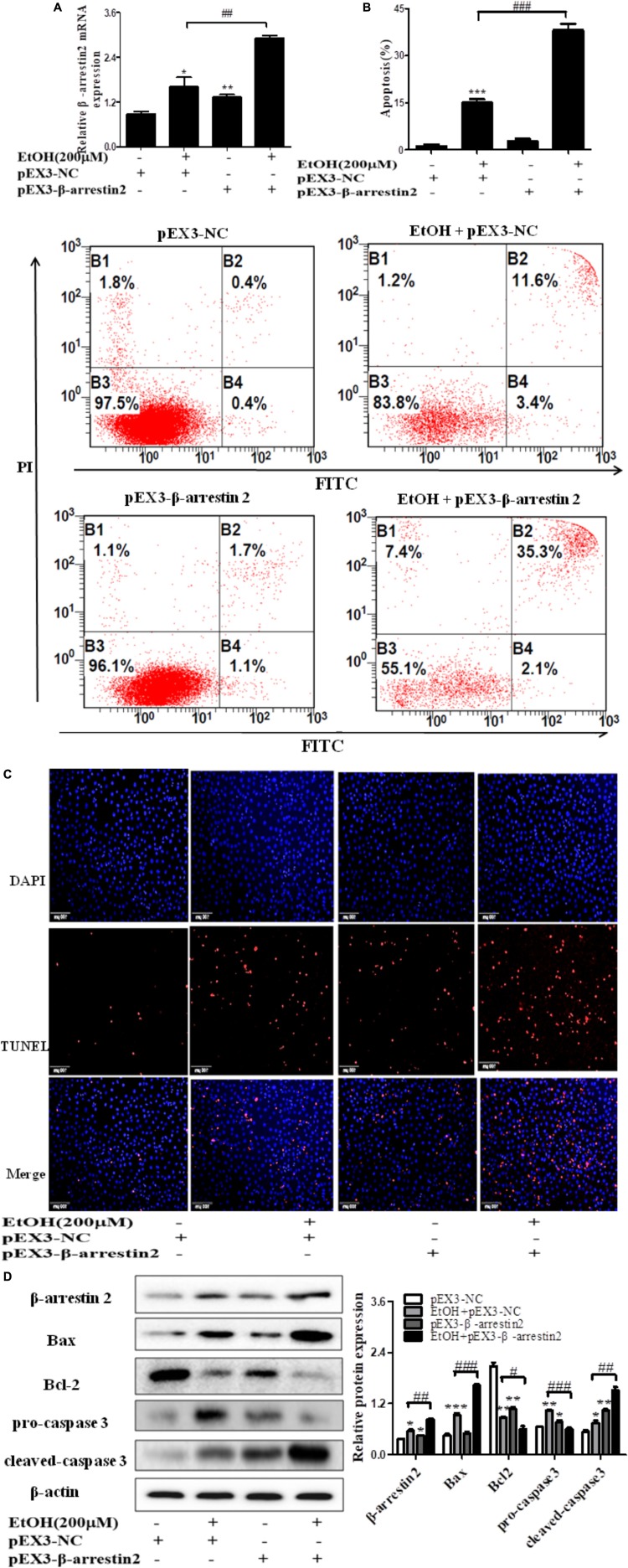
Effect of pEX3-Arrb2 on EtOH-stimulated AML-12 cells. **(A)** The Arrb2 levels were detected by real-time PCR after over-expression plasmid construction. The results are shown as relative expression against control expression without treatment. **(B)** Apoptosis of AML-12 cells were analyzed by flow cytometry with Annexin V-FITC and PI staining. **(C)** Representative images of TUNEL staining in different groups (original magnification, ×20). **(D)** The apoptosis relative protein expression were detected by western blot. A representative image of the three independent experiments were demonstrated. ^∗^*P* < 0.05, ^∗∗^*P* < 0.01, ^∗∗∗^*P* < 0.001 vs. pEX3-NC. ^#^*P* < 0.05, ^##^*P* < 0.01, ^###^*P* < 0.001 vs. EtOH + pEX3-NC.

To confirm the pro-apoptosis roles of Arrb2 in ALD, the experiment *in vitro* were carried out to further verify the results *in vivo* above. First of all, flow cytometric analysis combined with real-time PCR were used to find the optimal concentration of ethanol which can stimulate AML-12 cells to imitate acute alcohol treatment of ALD. The results in **Figures 4A,B** showed that ethanol enhanced mRNA levels of Arrb2 in a concentration-dependent manner and 200 μM of ethanol was selected as the optimal concentration. IF analysis further showed the changes of Arrb2 and the results in **Supplementary Figure 1A** showed a significantly increase in EtOH (200 μM)-stimulated group. Secondly, As shown in **Figure 5A**, Arrb2 siRNA was transfected into AML-12 cells with or without the treatment of ethanol transiently and the results of real-time PCR demonstrated that Arrb2 was successfully transfected into AML-12 cells because the mRNA levels was decreased obviously. In line with the above date *in vivo*, the hepatocyte apoptosis was substantially decreased by using TUNEL staining, flow cytometric analysis, and western blot when Arrb2 was blocked *in vitro* (**Figures 5B–D**). On the contrary, pEX3-Arrb2 was utilized to over-express Arrb2 and it was successfully up-regulated the mRNA levels of Arrb2 by real-time PCR (**Figure 6A**). Result showed that over-expression of Arrb2 led to significant hepatocyte apoptosis (**Figures 6B–D**).

### Arrb2 Promotes Hepatocyte Apoptosis by Inhibiting Akt Signaling Pathway

**FIGURE 7:**
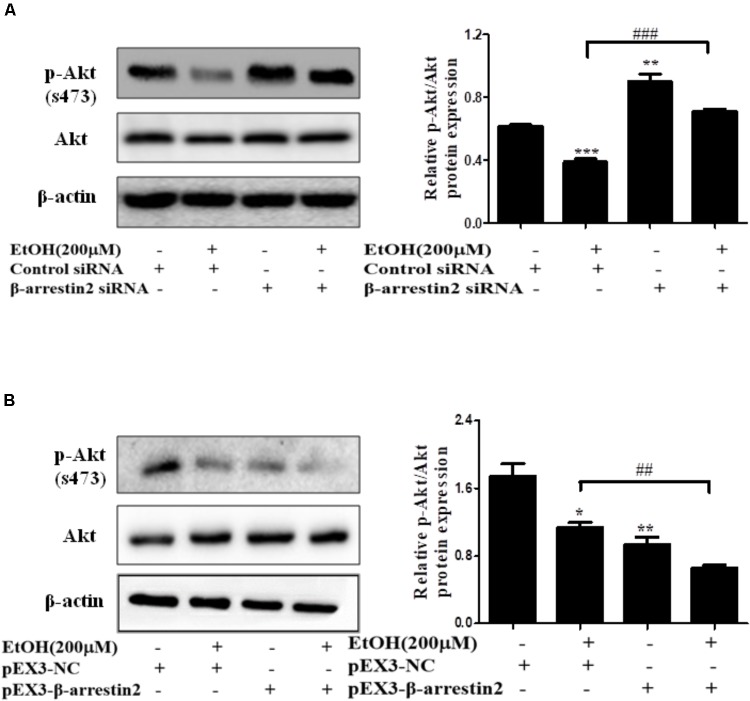
The interaction of Arrb2 with Akt in EtOH-stimulated AML-12 cells. **(A)** The protein levels of phospho-Akt in EtOH + Arrb2 siRNA transfected to AML-12 cells were assessed by Western blot. ^∗^*P* < 0.05, ^∗∗^*P* < 0.01, ^∗∗∗^*P* < 0.001 vs. Control siRNA. ^#^*P* < 0.05, ^##^*P* < 0.01, ^###^*P* < 0.001 vs. EtOH + Control siRNA. **(B)** The protein levels of phospho-Akt and in EtOH + pEX3-Arrb2 transfected to AML-12 cells were assessed by Western blot. ^∗^*P* < 0.05, ^∗∗^*P* < 0.01, ^∗∗∗^*P* < 0.001 vs. pEX3-NC. ^#^*P* < 0.05, ^##^*P* < 0.01, ^###^*P* < 0.001 vs. EtOH + pEX3-NC. Data shown are the mean ± SD from three independent experiments.

In recent years, evidence has shown that Arrb2 modulated Akt signaling pathway, but whether Akt signaling is associated with hepatocyte apoptosis in ALD remains unknown (Povsic et al., 2003; Beaulieu et al., 2005). To better understand the mechanism by which EtOH up-regulated Arrb2 expression in ALD and Arrb2 promoted hepatocyte apoptosis *in vivo* and *in vitro*, we next determined the protein levels of phospho-Akt in Arrb2-siRNA hepatocyte and pEX3-Arrb2 hepatocyte with or without the treatment of ethanol. Western blot results showed that Arrb2 increased hepatocyte apoptosis by inhibiting Akt signaling pathway (**Figures 7A,B**). To further confirm the effect of Akt signaling pathway on AML-12 cells apoptosis in ALD, a chemical inhibitor for Akt named LY249002 was used to suppress Akt pathway in EtOH-stimulated AML-12 cells. As shown in **Supplementary Figure 2**, results of western blot showed that treatment of 25 μM LY249002 significantly decreased p-Akt protein, leading to enhanced hepatocyte apoptosis.

## Discussion

Liver is the most important metabolic organ. Hepatocytes are damaged in response to the toxic metabolites, cytokines, chemokines, and reactive oxygen species (ROS) which cause cell apoptosis and death (Nassir and Ibdah, 2014). Alcohol abuse is a major cause for liver-related morbidity and mortality which can lead to steatosis, progressive fibrosis, cirrhosis, and ultimately HCC. Although the pathogenesis of ALD is poorly understand, several studies demonstrated that apoptosis of massive hepatocytes is a prominent feature of the initiation and progression stages of ALD (Ceni et al., 2014; Verma et al., 2016).

Available evidence suggested that apoptosis can either be triggered by intrinsic (mitochondria) or extrinsic (death receptor) pathway. The alcohol metabolism plays a key role in activation of mitochondria-dependent apoptotic pathway. Many apoptotic stimuli, such as DNA damage or growth factor, were involved in regulating mitochondria-dependent apoptotic pathway (Wang S. et al., 2016). Bailey and Cunningham (2002) demonstrated that the process of alcohol metabolism accelerated the flow of electrons and accumulated leakage of electrons in mitochondrial respiratory chain, thereby produced ROS. In addition, several studies revealed that alcohol exposure destroyed mitochondrial DNA resulting in the reduction of mitochondrial protein and ATP synthesis (Coleman and Cunningham, 1991). Furthermore, chronic alcohol exposure suppressed the rate of mitochondrial oxygen consumption, leading to increased sensitivity of hepatocytes in response to alcohol, and eventually caused the hypoxia and hepatocytes apoptosis or even death (Zelickson et al., 2011). It has been demonstrated that the mitochondria-dependent apoptotic pathway was mainly regulated by the Bcl-2 family proteins. Bcl-2-family members included three clusters: pro-survival subfamily (Bcl-2, Bcl-w, Bcl-XL, cl-1, and A1), pro-apoptotic subfamily (Bax, Bok, and Bak), and BH3-only group. The anti-apoptotic gene, Bcl-2, and the pro-apoptotic gene, Bax and caspase-3 played critical roles in ALD (Renault and Manon, 2011; Vervliet et al., 2016). It is now clear that caspases family became the main regulator in apoptosis and survival signals. In response to death signals, caspases are synthesized and processed to form active heterotetrameric enzymes (Wang S. et al., 2016). It was reported that activated caspase also cleaved pro-apoptotic Bcl-2 family protein (Yin and Ding, 2003).

In our study, the results showed that Arrb2 levels in liver tissue from EtOH-fed mice were notably higher than those in CD-fed mice. Furthermore, hepatocyte were isolated from the liver, and the results illustrated that the expression of Arrb2 was significantly increased in EtOH-fed mice compared to CD-fed mice. Arrb2 was also remarkably up-regulated in AML-12 cells in response to alcohol exposure. All above findings suggest that up-regulation of Arrb2 may play an important role in the development of ALD. However, the possible mechanisms for the EtOH-mediated upregulation of Arrb2 in ALD remains unknown. Arrb2 played an anti-inflammatory role of fenoterol in AICAR-treated THP-1 cells by activating nuclear transcription factor NF-κB (Wang W. et al., 2016). In systemic diseases, Arrb2 decreased the secretion of NF-κB-dependent gene products from fibroblast-like synoviocytes (Yu et al., 2015). Additionally, the bone marrow-derived macrophages from Arrb2 knockout mice showed a greater IκB kinase activity than WT mice macrophages (Liu et al., 2016). Therefore, we supposed that the nuclear transcription factor NF-κB may be involved in EtOH-mediated upregulation of Arrb2 and this possibility remained to be further explored. Then we further evaluated the function of Arrb2 in hepatocyte apoptosis. Our data demonstrated that knockdown of Arrb2 diminished hepatocyte apoptosis *in vivo.* In line with the *in vivo* data, the results in our study demonstrated that Arrb2 largely promoted apoptosis of hepatocyte *in vitro*.

It has been shown that the phosphatidylinositol 3-kinase (PI3K)/Akt pathway modulates cell survival, apoptosis, autophagy, and differentiation (Ouyang et al., 2017; Zhou et al., 2018). The mechanisms of PI3K/Akt/mTOR signaling pathway activation include the extension or mutation of PI3K and Akt, the loss of phosphatase and tensin homolog (PTEN) function and activation of growth factor receptor (LoPiccolo et al., 2008). The PI3K/Akt/mTOR signaling pathway is a prototypic survival pathway and is activated in many types of cancer disease (Mayer and Arteaga, 2016; Li et al., 2018). For example, the PI3K regulatory subunit p85 alpha can suppress tumor extension through negative regulation of growth factor signaling in breast cancers and HCCs. *N*-glycosylation of β4-integrin can promote tumor development and progression through activating PI3K in human cutaneous squamous cell carcinoma (Engelman, 2009; Taniguchi et al., 2010; Kariya et al., 2018). Consistently, activation or inhibition of PI3K/Akt/mTOR signaling pathway also played a critical role in many types of liver diseases. Zhang et al. (2018) demonstrated that long non-coding RNA lncARSR promoted hepatic lipogenesis by activating PI3K/Akt/mTOR pathways in non-alcoholic fatty liver disease (NAFLD). Conversely, Wei et al. (2018) revealed that asiatic acid attenuated HSC activation and extra cellular matrix (ECM) synthesis by inhibiting PI3K/Akt/mTOR signaling pathways in liver fibrosis (Zhang et al., 2018). In recent papers, it has been shown that Arrb1 and Arrb2 modulated Akt signaling pathway in HCC but it remains unknown that whether Akt signaling is associated with hepatocyte apoptosis in ALD (Yang et al., 2015; Yin et al., 2016). Given the above, the protein levels of phospho-Akt was detected in Arrb2 knockdown and overexpression hepatocytes with or without the treatment of ethanol. Our data showed that deficiency of Arrb2 increased phosphorylation of Akt while over-expressing Arrb2 suppressed Akt activation. In conclusion, our study is the first report in exploring the potential role of Arrb2 and the molecular mechanisms for the regulation of Arrb2 on hepatocyte apoptosis in ALD. This finding gained the support from the study that Arrbs can scaffold different molecules thereby playing different, even opposite effects on the same signaling pathway (Ma and Pei, 2007; Qi et al., 2016). More importantly, our study illustrates the cross-talk of Arrb2 with Akt signaling pathway and provides new insights of Arrb2 in hepatocyte apoptosis. Furthermore, Arrb2 may be used as a novel therapeutic target for ALD. However, the upstream modulators for Arrb2 in ALD remain to be further investigated.

## Author Contributions

JL contributed the experimental materials. Y-YS designed the experiments and research. Y-XZ performed the experiments. X-FL analyzed the data. CH and X-MM performed the experiments. All authors reviewed and approved this manuscript.

## Conflict of Interest Statement

The authors declare that the research was conducted in the absence of any commercial or financial relationships that could be construed as a potential conflict of interest.
