# Supplementary figures and images for: β-Arrestin 2 Promotes Hepatocyte Apoptosis by Inhibiting Akt Pathway in Alcoholic Liver Disease
Source: Front Pharmacol. 2018 Sep 19;9:1031. doi: 10.3389/fphar.2018.01031 (PMC6156347; doi:10.3389/fphar.2018.01031)

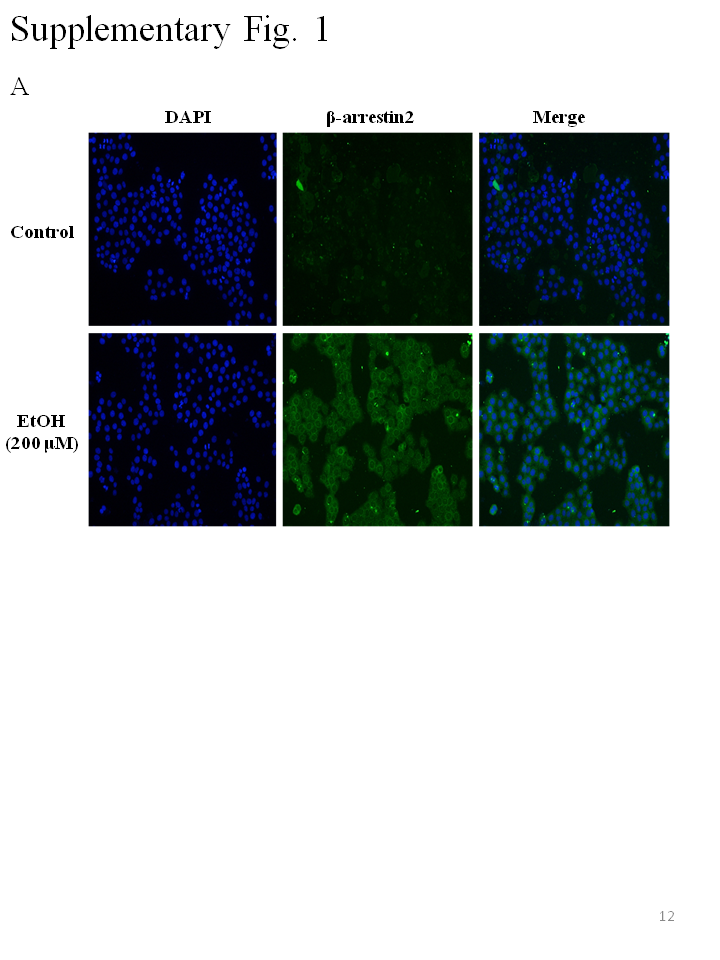

Supplement: Supplementary file 1 [file Image_1.tif]

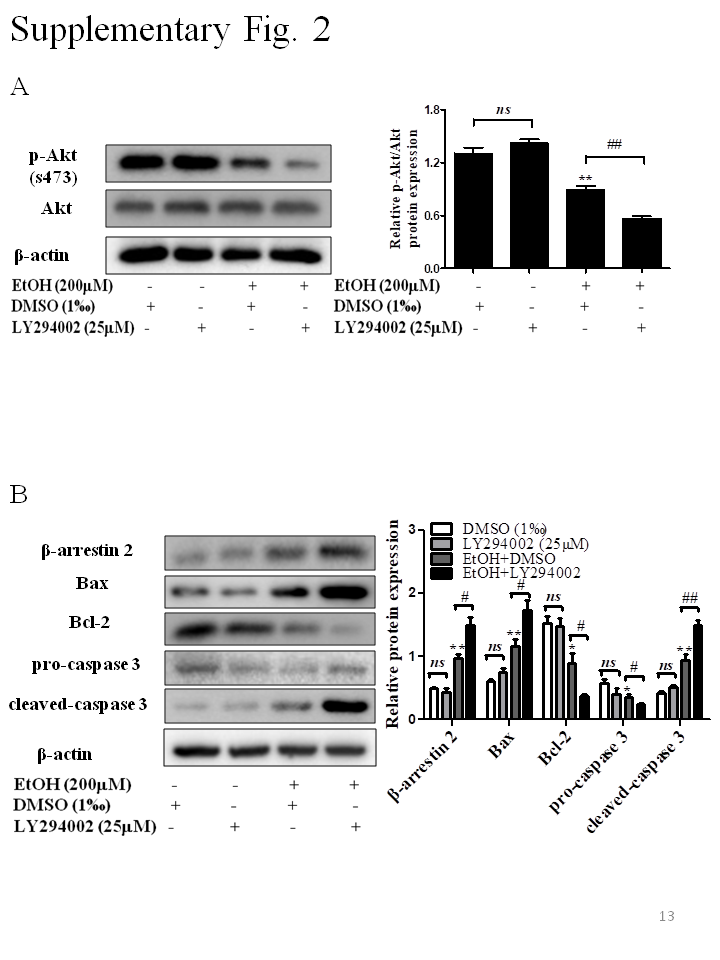

Supplement: Supplementary file 2 [file Image_2.tif]

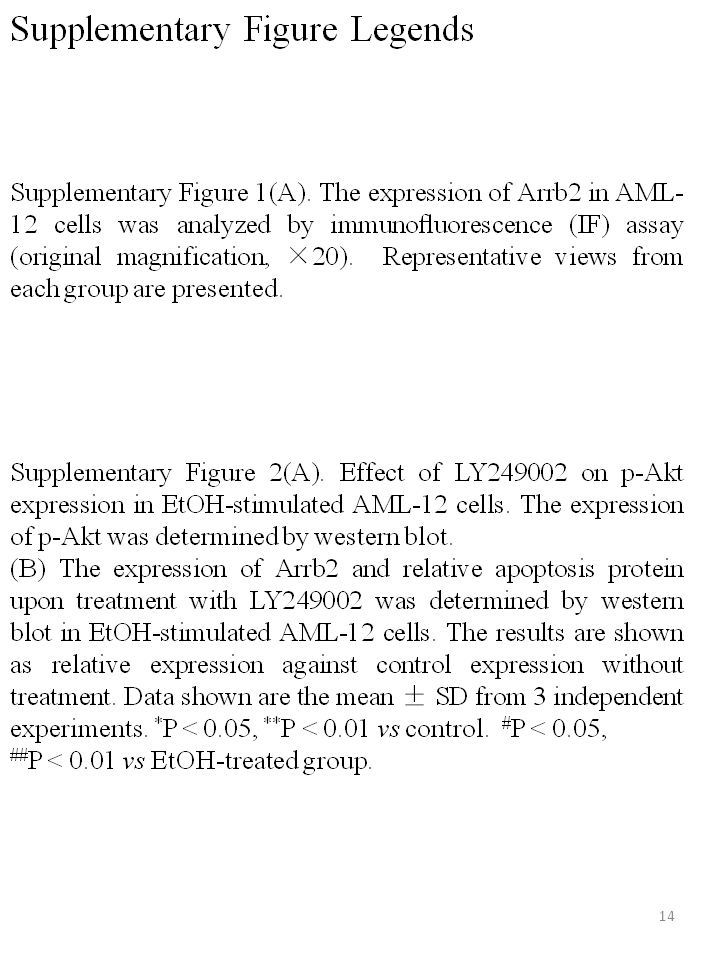

Supplement: Supplementary file 3 [file Image_3.tif]

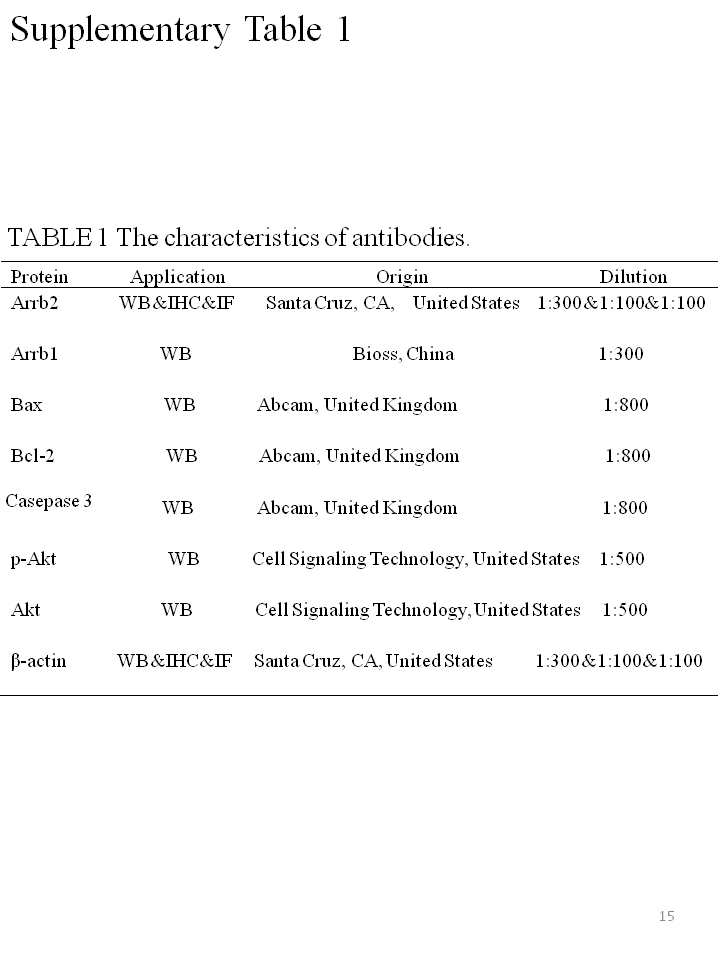

Supplement: Supplementary file 4 [file Image_4.tif]
